# Cortical synapses in acute hepatic encephalopathy: morphology and expression of proteins involved in synaptic transmission

**DOI:** 10.1186/2193-1801-4-S1-P39

**Published:** 2015-06-12

**Authors:** Mariusz Popek, Małgorzata Frontczak-Baniewicz, Barbara Zabłocka, Jan Albrecht, Magdalena Zielińska

**Affiliations:** Mossakowski Medical Research Centre Polish Academy of Sciences, Neurotoxicology, Poland

**Keywords:** Ammonia, Synapses, Synaptic proteins

Hepatic encephalopathy (HE) is manifested by impaired glutamatergic transmission. The current view is that HE spares the glutamatergic synapse, its dysfunction being primarily due to impaired astrocytic control. Here we show that in acute HE, the glutamatergic synapse by itself presents discrete changes in the ultrastructure (electron microscope) and expression of functionally critical synaptic proteins (Western blot). We used C57Bl6 mice subjected to a hepatotoxic insult in the azoxymethane (AOM) model. Cerebral cortex of AOM mice was ultrastrcturally characterized by the presence of increased numbers (~by 15%) of enlarged synapses showing abundance of synaptic vesicles in the presynaptic zone. The expression of synaptophysin and synaptotagmin in S2 fraction was increased by ~80% and ~30%, respectively. The expression of the NR1 subunit of NMDA receptor, but not of NR2A, was slightly increased. The amount of PSD-95 in P2 fraction and nNOS in S2 fraction, were elevated by ~40% and ~30% respectively. The expression of PKCζ was increased by ~30% and ~40% at the mRNA and protein level, respectively. PKCζ protein level was reduced by ~20% in P2 membrane fraction and elevated by ~30% in S2 cytosolic fraction suggesting its altered intracellular trafficking. This report is to our knowledge the first to demonstrate distinct changes in the synaptic ultrastructure and composition of synaptic proteins in the acute stage of HE. Except for PKCζ, all the other changes are indicative of a compensatory response of as yet unknown functional implications.

